# Shock Indices as Predictors of Outcomes in Emergency Department Patients With Emergency Severity Index Level 3

**DOI:** 10.7759/cureus.83379

**Published:** 2025-05-02

**Authors:** Sara Usman, Kamlesh M Bhojwani, Ahmed Raheem, Mehmood A Khan, Nadeem U Khan

**Affiliations:** 1 Emergency Medicine, Aga Khan University Hospital, Karachi, PAK; 2 Medicine, Aga Khan University Medical College, Karachi, PAK

**Keywords:** age shock index, emergency department, emergency severity index, modified shock index, shock index, shock indices

## Abstract

Background: The shock index (SI) and its derivatives, the modified shock index (MSI) and age shock index (ASI), have shown potential as prognostic tools in various clinical settings. However, their utility in predicting outcomes among Emergency Severity Index (ESI) level 3 emergency department (ED) patients remains understudied.

Objective: This study aims to evaluate the performance of SI, MSI, and ASI in predicting length of hospital stay (LOS), in-hospital mortality, intensive care unit (ICU) admission, and readmission rates in ESI level 3 ED patients.

Materials and methods: This prospective cross-sectional study enrolled 250 ESI level 3 patients from the ED, aged ≥20 years, at a tertiary care hospital in Pakistan, from August 1, 2021, to January 10, 2022. SI, MSI, and ASI were calculated from triage vital signs. Logistic regression analyses assessed associations between the indices and study outcomes. Predictive performance was evaluated using receiver operating characteristic (ROC) curves.

Results: All three indices exhibited significant independent associations with mortality, even after adjusting for confounders (SI ≥1.2, OR: 11.1; MSI ≥1.0, OR: 8.82; ASI ≥36.8, OR: 12.14). ASI remained independently associated with LOS (ASI ≥36.8, OR: 3.23). ROC analyses demonstrated good predictive ability for mortality (AUC 0.84 for SI, 0.82 for ASI) and ICU admission (AUC 0.81 for SI, 0.79 for ASI).

Conclusions: Among ESI level 3 ED patients, SI ≥1.2 demonstrated strong predictive value for mortality, while ASI ≥36.8 showed moderate predictive value and was additionally associated with longer LOS. These indices, particularly the SI and ASI, may be useful adjuncts to clinical assessment for predicting mortality risk in this patient population.

## Introduction

The shock index (SI) is a score that reflects a patient's hemodynamic status and is calculated by dividing the heart rate (HR) by the systolic blood pressure (SBP). In patients experiencing shock, an elevated HR and decreased SBP lead to an increased SI, which has been linked to increased mortality [1]. However, since SI only accounts for the SBP, it may underpredict the severity of underlying shock in certain patient subgroups.

Numerous studies across various clinical settings have demonstrated SI's adequate ability to predict mortality in conditions such as trauma, pneumonia, acute pulmonary embolism, stroke, and acute myocardial infarction [2-8]. SI has also proven useful as a marker for early recognition of sepsis [9]. Numerous studies have consistently shown that SI outperforms vital sign measurements alone in hospital settings, providing a more accurate assessment.

Two derivatives of SI, the modified shock index (MSI) and the age shock index (ASI), have been shown to enhance the predictive capabilities of SI. MSI is calculated by dividing the HR by the mean arterial pressure (MAP = (SBP + 2 × diastolic blood pressure (DBP)/3), accounting for diastolic and systolic pressure. Conversely, ASI incorporates the patient's age by multiplying it by the SI value [10,11]. While not as extensively studied as SI, these variants theoretically offer additional benefits by considering factors beyond HR and SBP.

Abnormal vital signs and indices can predict intensive care unit (ICU) admission and in-hospital mortality among adults triaged in the emergency department (ED) [12]. In a retrospective analysis of over 22,000 ED patients who received intravenous fluids, Liu et al. found that MSI was superior to standard SI in predicting mortality. However, multiplying ASI may provide better prognostic value in older adults with higher baseline blood pressure than SI alone [10].

Various triage systems are employed globally to prioritize ED patients based on clinical urgency and resource needs. The Emergency Severity Index (ESI), the most widely used triage system in the United States, is a five-level algorithm that categorizes patients based on illness severity and anticipated resource requirements [13]. ESI level 1 indicates life-threatening conditions, level 2 denotes high-risk features (e.g., altered mental status, severe pain, or abnormal vital signs), and levels 3, 4, and 5 are assigned to patients without immediate life threats, based on expected resource use [13-17]. In contrast, the National Early Warning Score 2, widely used in the United Kingdom, assigns scores based on physiological parameters (e.g., respiratory rate (RR), oxygen saturation (SpO₂), HR) to identify patients at risk of deterioration, primarily for in-hospital monitoring but increasingly applied in ED settings [18]. The Canadian Triage and Acuity Scale, used in Canada, also employs a five-level system but emphasizes time-to-physician assessment, with level 1 requiring immediate evaluation and level 5 indicating non-urgent conditions [19,20]. Similarly, the Australasian Triage Scale, used in Australia and New Zealand, prioritizes patients across five categories based on maximum waiting times, from immediate (category 1) to non-urgent (category 5) [19,20]. While these systems differ in their approach, the ESI was selected for this study due to its widespread adoption in our institution and its focus on acuity and resource allocation, which aligns with the study’s objectives in evaluating SI in ESI level 3 patients [13-17].

In resource-limited countries like Pakistan, where integrated care services are lacking and the healthcare system faces immense pressure, particularly in EDs that serve as the primary entry point for all patients, there is a critical need for simple, cost-effective, and bedside measures that can predict outcomes, guide initial management, and optimize appropriate resource allocation. The main challenge lies with ESI level 3 patients, who present with only mildly abnormal vital signs. For critically ill patients triaged as ESI level 1 or 2, and stable patients triaged as level 4 or 5, the associations between SI with mortality and ICU admission are well-established [14]. However, the relationship between these indices and outcomes may differ for the ESI level 3 population.

Most prior studies have focused primarily on mortality and ICU admission as key outcomes when evaluating SI, without examining potential associations with other significant outcome measures like length of hospital stay (LOS) and readmission rates. To address this gap and reduce the confounding effect of variable treatment intensities across different triage levels, our present study aims to determine the association of SI, MSI, and ASI in predicting a comprehensive set of outcomes, including LOS, mortality, ICU admission, and readmission rates, specifically among ESI level 3 patients. By focusing solely on this ESI level, we aim to provide valuable insights into the utility of these SI as prognostic tools for a challenging subgroup of ED patients.

## Materials and methods

Study design and setting

This cross-sectional study employed non-probability convenience sampling and was conducted at the department of emergency medicine of one of the tertiary care hospitals in Karachi, Pakistan, over a six-month period from August 1, 2021, to January 10, 2022.

Study population

The study included patients aged ≥20 years, who were assigned ESI level 3 at triage. Patients were initially assigned priority level 4 (P4) but upgraded to P3 due to deterioration of vital signs or clinical instability, those initially triaged as ESI level 1 or 2 and subsequently moved to ESI level 3 after stabilization, patients referred out to other institutions, those leaving against medical advice, patients with a code status of do not resuscitate or comfort care, and patients with a history of trauma were excluded from the study.

Data collection procedure

A total of 250 consecutive patients were enrolled in the study. An exemption from ethical approval was obtained from the Ethical Review Committee of Aga Khan University (approval number: 2020-5578-15252, approval date: January 4, 2021) prior to starting the data collection. The study was conducted during the COVID-19 pandemic, which imposed restrictions on data collection, allowing the team to collect data only on specific days and times. Patients categorized as ESI level 3 at triage, fulfilling the inclusion criteria, were recruited for this study. Prior to including the patients, the principal investigator obtained verbal informed consent from all patients. The data to be obtained from the triage slip by a registered nurse, blinded to the study objectives. On the way to finding a value that can be easily studied, there was a need to determine the threshold values for vital signs based on common cut-offs for SI. Since no standard threshold value was available for the use of SI, MSI, and ASI, we used the mortality curves for each of the three SI as a starting point by using the receiver operating characteristic (ROC) curve and determining the threshold value in terms of different sensitivities and specificities along with positive predictive value and negative predictive value. Patients were followed until discharged.

Study outcomes

Our study outcomes were LOS (primary outcome), in-hospital mortality, ICU admission, and readmission (secondary outcomes). Study variables were age, sex, BMI, HR, RR, temperature, SpO₂, SBP, DBP, MAP, SI, MSI, and ASI.

Statistical analysis

Data was entered and analyzed using SPSS Statistics version 21 (IBM Corp. Released 2012. IBM SPSS Statistics for Windows, Version 21.0. Armonk, NY: IBM Corp.) and R software (R Foundation for Statistical Computing, Vienna, Austria, https://www.R-project.org/). The normality assumption for continuous data was evaluated using the Shapiro-Wilk test. Normally distributed continuous variables were summarized as mean ± standard deviation (SD), while non-normal data were presented as median with interquartile ranges (IQR). Frequencies and percentages were reported for categorical variables. Comparisons between groups were made using the independent sample t-test, the Mann-Whitney U test for continuous variables, and the Pearson chi-square test or Fisher's exact test for categorical and nominal variables. Univariable and multivariable binary logistic regression analysis was performed for the scoring system variables (SI, MSI, and ASI) to identify potential associations with the primary and secondary outcomes. The performance characteristics of the scoring systems, including sensitivity, specificity, positive predictive value, negative predictive value, and accuracy, were calculated for mortality prediction. ROC curve analysis was conducted for the scoring systems (SI, MSI, and ASI) to assess their ability to predict all four outcomes, with the area under the curve (AUC) being obtained. A two-sided p-value of ≤0.05 was considered statistically significant.

## Results

The study included 250 ESI level 3 ED patients with a mean age of 43 ± 17 years and a slight predominance of females (56%) over males (44%). Hypertension (28.4%) and diabetes mellitus (20.8%) were the most prevalent comorbidities (Table 1).

**Table 1:** Patient demographics, baseline characteristics, and associations between study variables and outcomes including mortality, ICU admission, LOS, and readmission among ESI level 3 ED patients SBP: systolic blood pressure, DBP: diastolic blood pressure, MAP: mean arterial pressure, HR: heart rate, SI: shock index, MSI: modified shock index, ASI: age shock index, ICU: intensive care unit, BMI: body mass index, DM: diabetes mellitus, HTN: hypertension, CKD: chronic kidney disease, CVA: cerebrovascular accident, LOS: length of hospital stay, SD: standard deviation, ESI: Emergency Severity Index, SpO₂: oxygen saturation * p-value <0.05 is considered statistically significant

	Total	LOS			Mortality			ICU admission			Readmission		
		<3 days	≥3 days	p-value	Alive	Expired	p-value	Non-ICU	ICU	p-value	Yes	No	p-value
Total patients (n)	250	39	211	-	245	05	-	244	06	-	21	229	-
Age (years), mean ± SD	43 ± 17	39.72 ± 16.06	43.32 ± 17.54	0.234	43 ± 17	46 ± 15	0.696	43 ± 17	45 ± 13	0.775	48.33 ± 19.85	42.25 ± 17.05	0.124
Gender distribution, n (%)													
Male	110 (44%)	16 (14.5%)	94 (85.5%)	0.684	105 (95.5%)	05 (4.5%)	0.011*	104 (94.5%)	06 (5.5%)	0.005*	05 (4.5%)	105 (95.5%)	0.051
Female	140 (56%)	23 (16.4%)	117 (83.6%)		140 (100%)	0 (0%)		140 (100%)	0 (0%)		16 (11.4%)	124 (88.6%)	
LOS (hrs)	6.6 (43.9-4.2)	-			6.5 (36.4-4.2)	169.8 (210-157.9)	0.542	6.5 (36.4-4.2)	163.9 (210-68)	<0.001*	7.5 (49.7-5.2)	6.5 (42-4.2)	0.342
Triage vitals													
SBP (mmHg)	129 (144-117)	126 (141-114)	130 (144-118)	0.368	129 (144-117)	120 (130-119)	0.316	129 (144-117)	120 (130-108)	0.141	125 (134-116)	129 (144-117)	0.541
MAP (mmHg)	98 (106-89)	95 (109-89)	98 (106-89)	0.698	98 (106-89)	93 (97-88)	0.267	98 (107-89)	92 (97-88)	0.181	95 (100-86)	98 (108-89)	0.089
HR (beats/min)	91 (104-78)	85 (98-77)	92 (105-78)	0.048*	91 (104-78)	88 (110-82)	0.724	91 (104-78)	87 (110-82)	0.85	96 (104-80)	91 (104-78)	0.82
RR (breaths/min)	20 (20-20)	20 (18-21)	22 (19-24)	0.035*	20 (20-20)	24 (28-21)	0.001*	20 (20-20)	23 (28-20)	0.003*	20 (20-20)	20 (20-20)	0.518
Temperature (^0^C)	37 (37-36)	37 (37-36)	37 (37-37)	0.049*	37 (37-36)	37 (38-37)	0.064	37 (37-36)	37 (38-37)	0.129	37 (37-37)	37 (37-36)	0.562
SpO₂ (%)	99 (99-98)	99 (99-98)	99 (99-98)	0.133	99 (99-98)	93 (98-90)	0.013*	99 (99-98)	96 (99-90)	0.036*	99 (99-98)	99 (99-98)	0.888
Scoring systems													
SI	0.7 (0.8-0.6)	0.7 (0.8-0.6)	0.7 (0.9-0.6)	0.348	0.7 (0.8-0.6)	1.2 (1.2-0.8)	0.010*	0.7 (0.8-0.6)	1 (1.2-0.8)	0.010*	0.7 (0.8-0.6)	0.7 (0.8-0.6)	0.748
MSI	0.9 (1.1-0.8)	0.9 (1.1-0.8)	1 (1.1-0.8)	0.234	0.9 (1.1-0.8)	1.1 (1.1-0.9)	0.311	0.9 (1.1-0.8)	1 (1.1-0.9)	0.349	1 (1.1-0.8)	0.9 (1.1-0.8)	0.228
ASI	28.3 (35.7-21.4)	24.7 (34.4-18.5)	28.7 (36.7-22.2)	0.049*	28.1 (35.1-21)	40.6 (61.7-37)	0.014*	28 (35.1-20.9)	38.8 (61.7-31.9)	0.016*	32.9 (42.8-26.5)	27 (35.1-21)	0.073
Age groups													
1-50 years	178	29 (16.3%)	149 (83.7%)	0.635	176 (98.9%)	02 (1.1%)	0.12	175 (98.3%)	03 (1.7%)	0.246	12 (6.7%)	166 (93.3%)	0.137
51-80 years	72	10 (13.9%)	62 (86.1%)		69 (95.8%)	03 (4.2%)		69 (95.8%)	03 (4.2%)		9 (12.5%)	63 (87.5%)	
Comorbid conditions, n (%)													
HTN	71 (28.4%)	11 (15.5%)	60 (84.5%)	0.977	70 (98.6%)	01 (1.4%)	0.674	70 (98.6%)	01 (1.4%)	0.519	08 (11.3%)	63 (88.7%)	0.303
DM	52 (20.8%)	6 (11.5%)	46 (88.5%)	0.364	51 (98.1%)	01 (1.9%)	0.964	51 (98.1%)	01 (1.9%)	0.801	08 (15.4%)	44 (84.6%)	0.041*
Dyslipidaemia	08 (3.2%)	01 (12.5%)	07 (87.5%)	0.806	08 (100%)	0 (0%)	0.681	08 (100%)	0 (0%)	0.652	0 (0%)	08 (100%)	0.384
CKD	01 (0.4%)	0 (0%)	01 (100%)	0.667	01 (100%)	0 (0%)	0.886	01 (100%)	0 (0%)	0.875	0 (0%)	01 (100%)	0.762
CVA	03 (1.2%)	01 (33.3%)	02 (66.7%)	0.394	03 (100%)	0 (0%)	0.803	03 (100%)	0 (0%)	0.785	0 (0%)	03 (100%)	0.598
BMI (kg/m^2)^	24.5 (28-22.2)	24.5 (29.7-22.5)	24.5 (27.7-22)	0.765	24.5 (28.0-22.2)	23.4 (27.5-21.8)	0.788	24.5 (28.1-22.2)	22.7 (27.5-21.8)	0.537	25.3 (28.9-22.5)	24.4 (27.7-22)	0.381
Underweight (<18.5)	11	02 (18.2%)	09 (81.8%)	0.439	11 (100%)	0 (0%)	0.711	11 (100%)	0 (0%)	0.627	0 (0%)	11 (100%)	0.596
Normal (18.5 to 24.9)	121	20 (16.5%)	101 (83.5%)		118 (97.5%)	03 (2.5%)		117 (96.7%)	04 (3.3%)		09 (7.4%)	112 (92.6%)	
Overweight (25 to 29.9)	76	08 (10.5%)	68 (89.5%)		74 (97.4%)	02 (2.6%)		74 (97.4%)	02 (2.6%)		07 (9.2%)	69 (90.8%)	
Obese (≥30)	42	09 (21.4%)	33 (78.6%)		42 (100%)	0 (0%)		42 (100%)	0 (0%)		05 (11.9%)	37 (88.1%)	

Notably, the male sex was significantly associated with higher mortality (4.5% vs. 0% in females, p=0.011) and ICU admission rates (5.5% vs. 0%, p=0.005). Among triage vitals, RR and SpO₂ were strongly linked to mortality (RR: p=0.001; SpO₂: p=0.013) and ICU admission (RR: p=0.003; SpO₂: p=0.036), highlighting their potential as early warning signs in this population. Additionally, patients with longer LOS (≥3 days) tended to have higher RR and slightly elevated temperatures at triage, indicating that these vital signs may reflect underlying conditions contributing to prolonged hospitalization.

The SI demonstrated distinct predictive patterns. SI and ASI were significantly associated with mortality (SI: p=0.010; ASI: p=0.014) and ICU admission (SI: p=0.010; ASI: p=0.016), underscoring their utility in identifying ESI level 3 patients at risk of severe outcomes. ASI correlated with longer LOS (p=0.049), indicating its broader prognostic value across multiple outcomes. Comorbidities like diabetes mellitus were associated with readmission (p=0.041), but other conditions (e.g., hypertension, chronic kidney disease) showed no significant relationships.

Univariate analysis

Univariate logistic regression analysis highlighted distinct patterns in the ability of SI, MSI, and ASI to predict clinical outcomes among ESI level 3 ED patients (Table 2).

**Table 2:** Univariate logistic regression analysis for predicting mortality, ICU admission, LOS, and readmission SI: shock index, MSI: modified shock index, ASI: age shock index, ICU: intensive care unit, LOS: length of hospital stay, OR: odds ratio, CI: confidence interval * p-value <0.05 is considered statistically significant

	LOS		Mortality		ICU admission		Readmission	
	OR (95% CI)	p-value	OR (95% CI)	p-value	OR (95% CI)	p-value	OR (95% CI)	p-value
SI ≥1.2	1.6 (0.86-2.99)	0.139	4.26 (0.8-52.6)	0.214	5.39 (0.97-30.14)	0.055	0.6 (0.24-1.53)	0.286
MSI ≥1.0	2.59 (1.27-5.3)	0.009*	2.46 (1.21-18.9)	0.042*	5.6 (1.09-28.79)	0.039*	0.58 (0.2-1.68)	0.313
ASI ≥36.8	3.51 (1.87-6.6)	<0.001*	8.19 (1.03-58.9)	0.049*	6.56 (1.17-36.76)	0.032*	0.79 (0.29-2.14)	0.643

For LOS, both MSI (≥1.0) and ASI (≥36.8) were significantly associated with prolonged LOS (OR=2.59 and OR=3.51, respectively), while SI (≥1.2) showed no significant relationship. This suggests that incorporating diastolic pressure (MSI) or age (ASI) may better capture factors influencing hospitalization length compared to SI alone.

For mortality, MSI and ASI again demonstrated significant predictive value. ASI showed the strongest association (OR=8.19), indicating its potential as a marker for identifying patients at risk of fatal outcomes in this population. SI’s association with mortality was non-significant in univariate analysis, contrasting with its performance in adjusted models. This highlights the potential confounding role of age and hemodynamic stability in mortality risk.

For ICU admission, MSI and ASI were significant predictors (OR=5.6 and OR=6.56, respectively). None of the indices predicted readmission, implying that other factors beyond these SI may influence the likelihood of patients returning to the hospital after discharge.

Multivariate analysis

The adjusted multivariate analysis revealed important insights about the independent predictive value of SI after accounting for potential confounders (Table 3).

**Table 3:** Multivariate logistic regression analysis for predicting mortality, ICU admission, and LOS SI: shock index, MSI: modified shock index, ASI: age shock index, ICU: intensive care unit, LOS: length of hospital stay, OR: odds ratio, CI: confidence interval * p-value <0.05 is considered statistically significant

Factors	LOS		Mortality		ICU admission		Readmission	
	OR (95% CI)	p-value	OR (95% CI)	p-value	OR (95% CI)	p-value	OR (95% CI)	p-value
SI ≥1.2	0.74 (0.3-1.82)	0.511	11.1 (2.38-63.4)	0.026*	2.4 (0.26-21.89)	0.437	0.71 (0.21-2.42)	0.587
MSI ≥1.0	2.53 (0.98-6.91)	0.051	8.82 (1.48-46.8)	0.018*	2.17 (0.27-17.67)	0.470	0.76 (0.19-3.1)	0.706
ASI ≥36.8	3.23 (1.68-6.23)	<0.001*	12.14 (3.24-19.4)	0.013*	4.55 (0.76-27.08)	0.096	0.91 (0.32-2.54)	0.851

For LOS, ASI ≥36.8 remained independently associated, with 3.23 times higher odds of longer LOS (p<0.001), possibly due to the influence of age on recovery and disease severity. In contrast, neither SI nor MSI maintained a significant independent association with LOS, indicating that other clinical factors may confound their predictive value for this outcome.

For mortality, all three indices, SI ≥1.2 (OR=11.1; p=0.026), MSI ≥1.0 (OR=8.82; p=0.018), and ASI ≥36.8 (OR=12.14; p=0.013) remained independently associated with increased odds of mortality, with ASI again showing the most pronounced effect. This reinforces the potential of these indices, particularly ASI, as reliable tools for identifying patients at heightened risk of death, even after accounting for other variables.

Regarding ICU admission, none of the scoring systems were independently associated with increased odds of ICU admission in the multivariate analysis. This suggests that decisions for ICU care in this population may be driven by factors beyond these hemodynamic indices, such as clinical judgment or resource availability.

As with the univariate analysis, none of the indices predicted readmission, indicating that these tools may not adequately capture the complex interplay of factors leading to hospital readmissions, such as post-discharge care or social determinants of health.

ROC curve analysis

**Figure 1:**
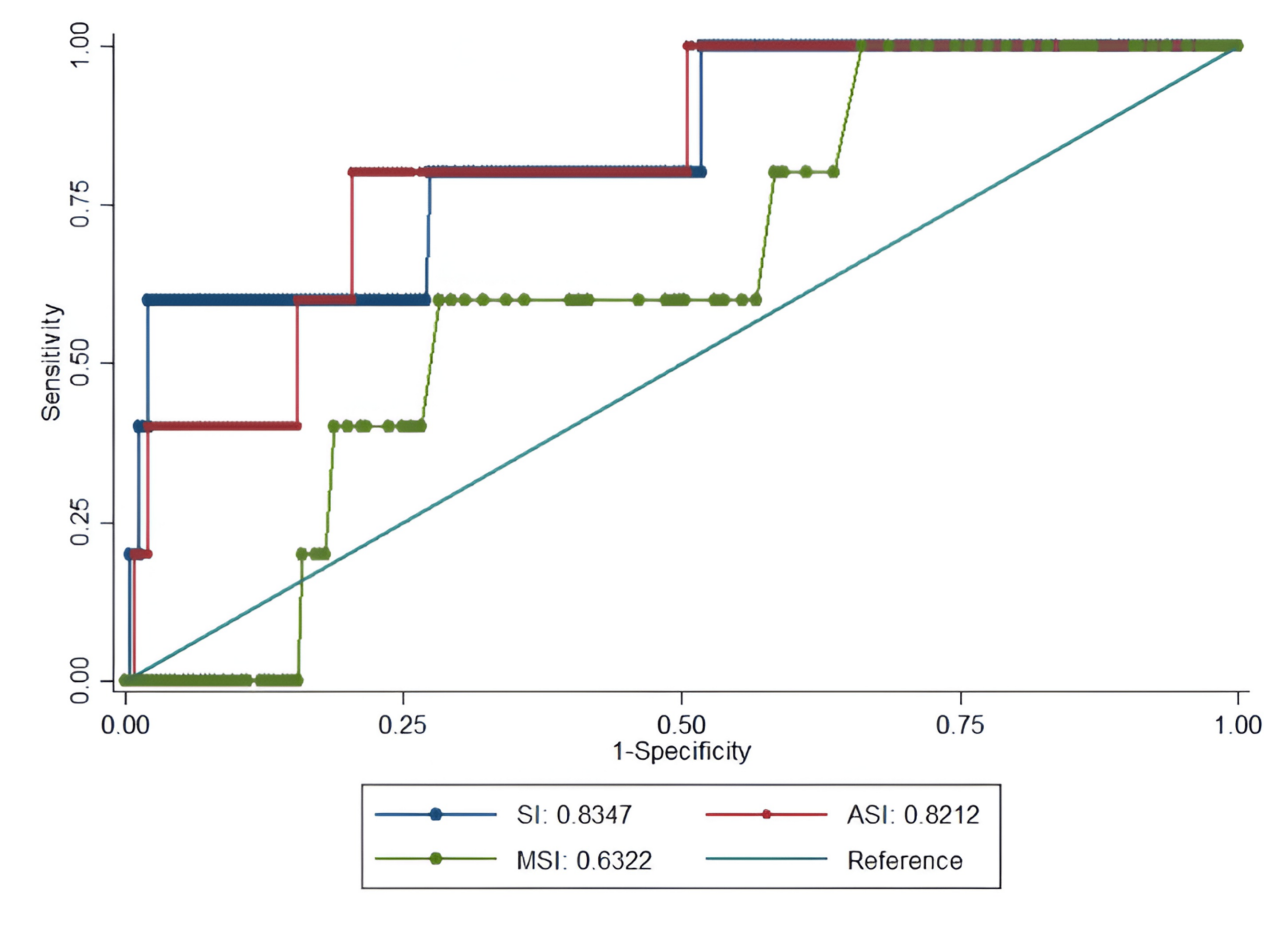
ROC curves for predicting in-hospital mortality among ESI level 3 ED patients using SI Comparison of predictive performance between SI (blue), MSI (red), and ASI (green) for in-hospital mortality. The dashed diagonal line represents reference (AUC=0.5). ASI demonstrated the best balance of sensitivity and specificity (AUC=0.82). ROC: receiver operating characteristic, ESI: Emergency Severity Index, ED: emergency department, SI: shock index, MSI: modified shock index, ASI: age shock index, AUC: area under the curve

**Figure 2:**
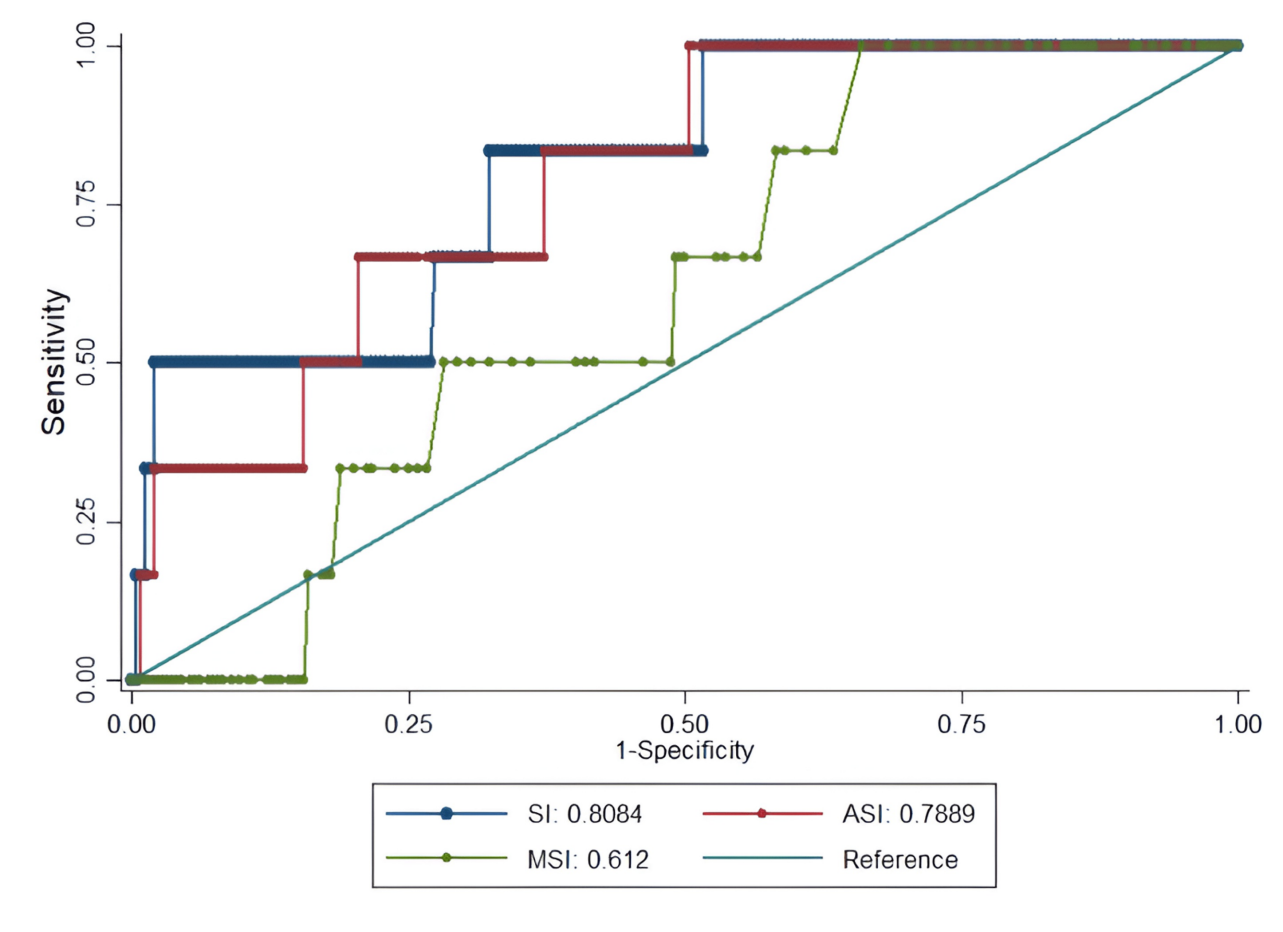
ROC curves for predicting ICU admission in ESI level 3 ED patients using SI Predictive performance of SI (blue), MSI (red), and ASI (green) for ICU admission. SI showed the highest discriminative ability (AUC=0.81). MSI and ASI achieved 100% sensitivity but with low specificity. ROC: receiver operating characteristic, ESI: Emergency Severity Index, ED: emergency department, SI: shock index, MSI: modified shock index, ASI: age shock index, AUC: area under the curve

**Figure 3:**
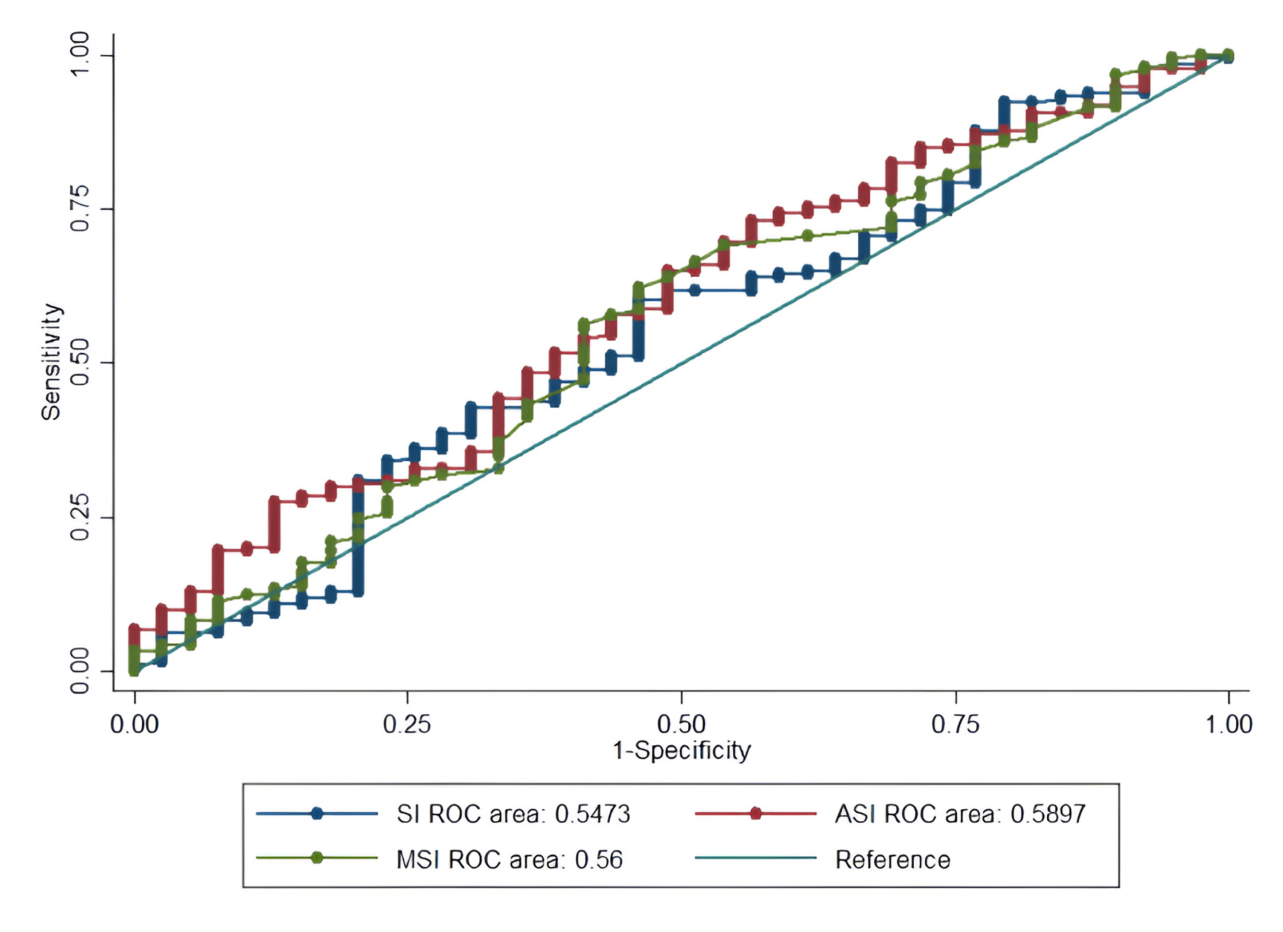
ROC curves for predicting prolonged LOS in ESI level 3 ED patients using SI Performance of SI in predicting prolonged hospitalization (≥3 days). All indices showed limited predictive value (AUC=0.55-0.59), suggesting they are unreliable standalone tools for this outcome. ROC: receiver operating characteristic, LOS: length of hospital stay, ESI: Emergency Severity Index, ED: emergency department, SI: shock index, MSI: modified shock index, ASI: age shock index, AUC: area under the curve

**Figure 4:**
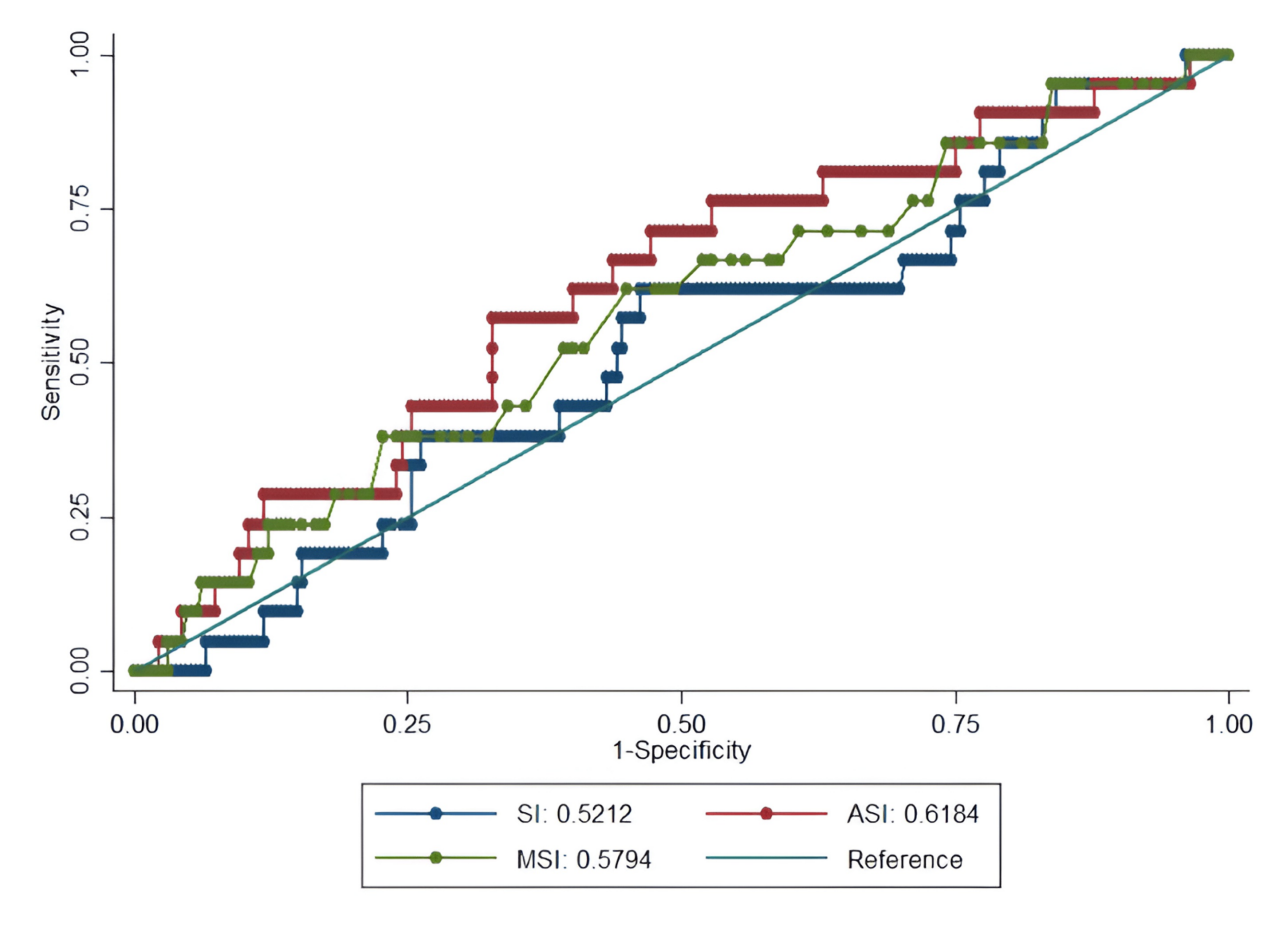
ROC curves for predicting 30-day hospital readmission in ESI level 3 ED patients using SI Predictive performance for readmission was poor across all indices (AUC=0.52-0.62), indicating that the SI have limited utility in identifying patients at risk of readmission. ROC: receiver operating characteristic, ESI: Emergency Severity Index, ED: emergency department, SI: shock index, MSI: modified shock index, ASI: age shock index, AUC: area under the curve

The ROC curve analysis provides critical insights into the clinical utility of SI as a predictive tool (Figures 1-4, Table 4).

**Table 4:** Performance of the SI, MSI, and ASI in predicting mortality, ICU admission, hospital LOS, and readmission, as assessed by ROC curve analysis SI: shock index, MSI: modified shock index, ASI: age shock index, ICU: intensive care unit, AUC: area under the curve, LOS: length of stay, ROC: receiver operating characteristic, LR+: positive likelihood ratio

Indicators	Threshold	AUC (95% CI)	Sensitivity	Specificity	LR+	Accuracy
	Value		(95% CI)	(95% CI)		
Mortality						
SI	1.2	0.84 (0.64-0.98)	60 (23.1-88)	98 (95.1-99.2)	29.4	0.97
MSI	1.0	0.63 (0.43-0.84)	98.6 (50.6-99.6)	33.9 (28.2-40.8)	1.5	0.39
ASI	36.8	0.82 (0.64-0.96)	80 (35.9-97.5)	79.6 (74.1-84.2)	3.9	0.8
ICU admission						
SI	0.79	0.81 (0.63-0.98)	83.2 (76.2-89.4)	67.6 (62.3-75.3)	2.5	0.68
MSI	0.83	0.61 (0.44-0.79)	100 (55.2-100)	34 (28.4-40.2)	1.5	0.36
ASI	27.02	0.79 (0.63-0.95)	100 (55.2-100)	49.6 (43.4-55.8)	2	0.51
Hospital stay						
SI	0.89	0.55 (0.49-0.68)	58.7 (51.2-66.5)	58 (49.3-63.4)	1.27	0.58
MSI	0.96	0.56 (0.45-0.66)	63.9 (52.8-72.1)	51.2 (46.6-57.4)	1.3	0.62
ASI	0.79	0.59 (0.49-0.68)	64.9 (58.2-69.7)	51.8 (46.7-63.4)	1.33	0.63
Readmission						
SI	0.71	0.52 (0.43-0.62)	61.9 (53.2-67.3)	53.7 (46.1-59.9)	1.33	0.54
MSI	0.83	0.58 (0.44-0.70)	60.3 (51.2-65.3)	51.3 (42.1-68.9)	1.23	0.53
ASI	31.01	0.62 (0.49-0.75)	62 (52-67)	59.8 (41.3-69.3)	1.5	0.6

Both SI and ASI demonstrated good predictive ability for mortality, with AUCs of 0.84 and 0.82, respectively. The optimal SI cutoff (≥1.2) showed high specificity (98%) but moderate sensitivity (60%), making it particularly valuable for ruling in high-risk patients. In contrast, ASI (≥36.8) offered a better balance between sensitivity (80%) and specificity (79.6%), suggesting it may be more versatile for clinical use (Figure 1).

For ICU admission, SI again performed well (AUC=0.81), with a lower threshold (≥0.79) showing good sensitivity (83.2%) but modest specificity (67.6%). MSI showed perfect sensitivity (100%) but poor specificity (34%), limiting its practical value (Figure 2).

The indices showed weaker performance for LOS and readmission prediction (AUC=0.52-0.62), confirming that their primary utility lies in acute risk assessment rather than resource use or post-discharge outcomes (Figures 3-4).

Table 4 summarizes the performance of SI, MSI, and ASI in predicting various outcomes, as determined by ROC curve analysis. It provides the AUCs, sensitivities, specificities, and accuracy for predicting LOS, mortality, ICU admission, and readmission.

## Discussion

This study evaluated the performance of three scoring systems, SI, MSI, and ASI, in predicting important clinical outcomes in ED patients. Notably, all three indices remained independently associated with increased mortality risk after adjusting for potential confounders, backing previous studies highlighting their utility as prognostic tools in various clinical settings [2-9]. Specifically, the predictive ability observed for ASI and SI aligns with the results reported by Torabi et al. in a similar ESI level 3 patient population [14]. However, our finding that MSI performed relatively poorly for mortality prediction contrasts with Liu et al.'s study, which suggested MSI as a better predictor of mortality [10]. These discrepancies may be attributable to differences in study populations and settings, underscoring the need for further validation across diverse cohorts.

The enhanced performance of ASI compared to MSI for mortality prediction is noteworthy. This observation aligns with prior research suggesting that incorporating age into the ASI calculation may confer added prognostic value, particularly in older patient populations [10,11]. Torabi et al. also reported age and SBP as independent predictors of mortality in ESI level 3 patients [14], further substantiating the potential advantages of age-adjusted indices like ASI.

Regarding predictions for ICU admission, the independent associations of MSI and ASI did not reach statistical significance in our multivariate model. This aligns with Torabi et al.'s study of ESI level 2 patients, where none of the SI were associated with ICU admission [21]. A potential explanation for this could be the multifactorial decision-making process involved in ICUs, which may rely on factors beyond just SI values alone.

ASI was the only index that maintained a statistically significant independent association with LOS, indicating its potential usefulness in predicting this important outcome measure. This finding is particularly noteworthy, as LOS is a critical metric for resource allocation and cost management in healthcare. However, previous studies evaluating SI have not extensively explored it.

The ROC curve analyses further backed the predictive performance of these indices, particularly SI and ASI, for mortality. The optimal cut-off values identified in our study can serve as useful reference points for clinicians when interpreting these indices in the ESI level 3 patient population. However, it is important to note that while the SI demonstrated moderate predictive capabilities, their performance was not exceptional, as evidenced by the modest AUC values. This could be due to the complex interplay of factors influencing patient outcomes, highlighting the need for a holistic approach to risk stratification rather than relying solely on these indices.

Our study contributes to the existing literature by specifically focusing on the ESI level 3 population, a subgroup that has been relatively understudied in the context of SI. By identifying the predictive value of these indices within this group, our findings can inform clinical decision-making and resource allocation strategies in the ED setting.

Strengths and limitations

A notable strength of our study is its prospective design and the inclusion of a comprehensive set of outcomes, encompassing not only mortality and ICU admission but also LOS and readmission rates. This holistic approach provides a more complete understanding of the utility of SI in predicting various clinical outcomes. However, our research is not without limitations. First, it was conducted at a single tertiary care center, which may limit the generalizability of our findings to other healthcare settings with different patient populations and resource constraints. Moreover, the study was conducted during the COVID-19 pandemic, which restricted data collection, allowing us to collect data only on specific days and times. These constraints may have contributed to a smaller sample size. While our sample size was adequate for the primary analyses, it may have limited our ability to detect more subtle associations, particularly in the multivariate models. Furthermore, we did not account for potential confounding factors, such as comorbidities or disease severity scores, which could have influenced the observed associations between the SI and outcomes. Future studies should consider incorporating these factors into their analyses to provide a better understanding of the predictive capabilities of these indices.

## Conclusions

This study highlights the potential utility of ASI and SI as predictive tools for mortality and other important outcomes in ED patients. ASI demonstrated the best overall performance. SI (≥1.2) demonstrated strong predictive value for mortality (positive likelihood ratio: 29.4), while ASI showed moderate predictive ability. While no single scoring system excelled across all evaluated outcomes and should not be used as a sole decision-making tool, they can serve as valuable adjuncts to clinical assessment and contribute to risk stratification strategies in this challenging patient population.

Future research should explore the integration of these SI with other prognostic factors and clinical decision support tools to enhance their predictive accuracy and utility in the ED setting. Additionally, multicenter studies with larger sample sizes are warranted to further validate and refine the optimal cut-off values for these indices in diverse patient populations.
